# A review on ozone therapy in periodontitis

**DOI:** 10.6026/97320630018634

**Published:** 2022-07-31

**Authors:** P Swarna Meenakshi, Arvina Rajasekar

**Affiliations:** 1 Department of Periodontics, Saveetha Dental College and Hospitals, Saveetha Institute Of Medical and Technical Sciences Chennai 600077, Tamilnadu, India

**Keywords:** Ozone, peri implantitis, periodontitis, biofilm

## Abstract

The biologic process of osseointegration (bone to implant interface), in which the implant forms an intimate relationship with the bone, provides the foundation for modern dental implants. Osseointegration necessitates a varying length of healing time. Despite the high success and survival rates of dental implants, problems do occur, necessitating ongoing periodontal and prosthodontic care. This failure frequently results in "peri-implantitis," which affects the soft and hard tissues surrounding the osseointegrated implants, resulting in the formation of a peri implant pocket and bone loss. Decontamination is a difficult feature of surgical regenerative therapy for peri-implantitis that has an impact on its success. Because microbial biofilms play such a significant part in the aetiology of peri-implant illnesses, it has long been assumed that eliminating microbial pathogens would be beneficial.

## Background:

Periodontitis is a destructive inflammatory illness of the teeth's supporting tissues that is caused by one or more microorganisms, resulting in gradual loss of the periodontal ligament and alveolar bone, as well as periodontal pocket formation, gingival recession, or both [1]. These changes are associated with pathologic tooth migration [2]. Among the various types of pathological migration studied like diastema, crowding, spacing, mesial tilt and distal tilt, it was found that spacing between the teeth (53 sites) was the most common and the distal tilt of the tooth (5 sites) was the least common type of pathologic migration [2].Inflammation and bone loss are hallmarks of periodontal disease (PD) [3]. Bacteria are the primary cause of periodontal disease, and more than 500 different bacterial species are thought to be capable of colonising the adult mouth [3]. Oral lesions have a significant impact on the quality of life of patients with advanced disorders [4]. Periodontitis has been implicated as an etiological or modulating factor in cardiovascular and cerebrovascular disease, diabetes, respiratory disease, and poor pregnancy outcomes, with several mechanisms proposed to explain this [5].

## History:

Christian Friedrich Schonbein was the first to note the advent of a pungent gas with an electric odour in 1839. He termed it ozone in Greek, and at the Basel Natural Science Society, he gave a talk titled "On the smell at the positive electrode during electrolysis of water" [6].

## Structure:

Ozone (O3) is a three-atom molecule that contains three oxygen atoms. It has a molecular weight of 47.98 g/mol and is a thermodynamically instable molecule that decomposes to pure oxygen with a short half-life depending on system variables such as temperature and pressure. Ozone is 1.6 times denser and ten times more soluble in water than oxygen (49.0 mL in 100 mL water at 0°C) [6]. The internal steric barrier in ozone's structure prevents it from creating a triangle configuration. As a result, instead of the predicted double bonds, each oxygen atom forms a single bond with another oxygen atom, leaving the ozone molecule with a negative charge.

## Properties:

At ambient temperature, ozone is a colourless gas with a strong odour, noticeable at concentrations as low as 0.02–0.05 ppm. 9 Its half-life changes as the temperature changes. It has a half-life of 40 minutes at 20 degrees Celsius and 140 minutes at 0 degrees Celsius [6].

## Ozone production:

Under the influence of variables such as UV radiation (from the sun) and electrical discharges, oxygen molecules in the air mix (lightning). Intense physical stress on water (such as in waterfalls and ocean waves slamming against rocks) leads to the creation of ozone in nature [6]. Ozone is produced by extremely specialised gazettes termed as Ozone Generators for medicinal purposes. High voltage tubes with outputs ranging from 4000 V to 14000 V are used to deliver medical grade oxygen. Ultraviolet light bulb, Corona discharge, or Cold plasma is the three principles used by ozone generators [7]. The heal ozone15 and ozotop are two popular ozone units in dentistry [7].

## Mechanism of action:

Because of its unique qualities, such as antibacterial, immuno stimulant, analgesic, antihypnotic, detoxicating, bioenergetic, and biosynthetic effects, ozone treatment has a wide variety of uses in treating many disorders [8].

## Antimicrobial action:

Ozone causes inactivation of bacteria, viruses, fungi, yeast and protozoa. It disrupts the integrity of the bacterial cell envelope by oxidation of phospholipids and lipoproteins [8]. Ozone at low concentration of 0.1 ppm is sufficient to inactivate bacterial cells including their spores. In fungi, O3 inhibits cell growth at certain stages, budding cells being the most sensitive. With viruses, the O3 damages the viral capsid and upsets the reproductive cycle by disrupting the virus-to-cell contact with peroxidation [9].

## Stimulation of oxygen metabolism:

The rate of glycolysis in red blood cells increases as a result of ozone treatment. This causes 2,3-diphosphoglycerate to be stimulated, resulting in an increase in the quantity of oxygen delivered into the tissues. Ozone stimulates ATP synthesis by boosting oxidative carboxylation of pyruvate in the Krebs cycle. It also causes a significant reduction in NADH and helps to oxidize cytochrome C. There is stimulation of production of enzymes which act as free radical scavengers and cell-wall protectors: glutathione peroxidase, catalase and superoxide dismutase and prostacycline, a vasodilator [9].

## Activation of immune system:

Ozone administered at a concentration of between 30 and 55 lg/cc causes the greatest increase in the production of interferon and the greatest output of tumour necrosis factor and interleukin-2 that launches an entire cascade of subsequent immunological reactions.

## Mechanism of action of O3 in human lung:

On average, ozone causes a significant reduction in critical ability. It raises mean specific airway resistance significantly while having no effect on peak transpulmonary pressure. It also increases respiratory rate while decreasing tidal volume [10].

## Modes of ozone administration:

The European Cooperation of Medical Ozone Societies warns from direct intravenous injections of ozone/oxygen gas that should not be practiced due to the possible risk of air embolism.

## Ozone gas application:

Ozone is created by converting oxygen to ozone. After that, the ozone is directed to a hand piece with a silicone cup. Differently designed silicone cups are available that prevent ozone from escaping between the silicone cup and the carious portion of the tooth [11]. For a minimum of 10 seconds, ozone is fed via the silicone cup over the tooth. The gadget collects the ozone in the silicone cup and converts it to oxygen.

## Ozone aqueous solution:

The following properties of ozone are used in this case:

[1] Disinfectant and sterilizing effect

[2] Hemostatic effect, especially in cases of haemorrhages

[3] Accelerated wound healing, improved oxygen supply and support of metabolic processes [11].

## Ozonated oil:

Ozonated oil as an adjunct to scaling and root planning in aggressive periodontitis was effective and studies shows that ozonated oils were effective for bone integration and increases the bone density [12].

## Commercial products:

### Heal ozone:

It is an air-based system in which gas is applied in a closed circuit. Its surplus is sucked out and manganese ions neutralize it. [12] The concentration of ozone is 2100 ppm in the cap adjacent to the tissue. Perfect air tightness of the cap is the necessity for the application of ozone.

### Ozonytron:

This device utilises the power of high frequency and voltage. Adjusting the ozone concentration in five levels is possible with the existing strength. A glass probe made of a combination of noble gases conducts and emits electromagnetic radiation is formed by a double glass camera. As the probe's tip makes contact with the body, energy is released surrounding the treated region, causing diatomic oxygen in the environment to split into solitary atomic oxygen and ozone [12]. The operation field has an ozone concentration of 10-100 g/ml. Because there is no closed circuit here, ozone might be used to treat difficult-to-reach and inaccessible locations like gingival pockets or root canals.

### Product photo:

Prozone is easy to use and safe to apply as the tissue compatible dosages can be preset according to the indication areas of periodontitis [12]. A hygienic procedure is ensured during the gassing of the pockets as the plastic attachments (Perio tips or Endo tips) are exchangeable.

### Ozotop:

It's a small, easy-to-use tabletop ozone generator with a free-flow ozone delivery mechanism that uses corona discharge. It was possible to quickly enter root canals and periodontal pockets. [12] In this device, ambient air is filtered and dried before being passed over a ceramic plate, where high voltage is then applied, resulting in the production of ozone. Due to the open nature of the system, a large volume of suction is necessary. Ozone is sprayed at 6, 12, 18, or 24 seconds depending on the treatment requirement (e.g., 12 s - surgical disinfection, 18 s - periodontal disinfection) [12].

### Ozone nano bubble water:

Because ozonated water has a half-life of around 20 minutes before degrading back into oxygen, its effectiveness must be ensured by utilising it within the first 5-10 minutes after creation. In 2008, CHIBA and TAKAHASHI created ozone Nano bubble water (NBW3) to address this issue. A micro-bubble (50 m in diameter) in an electrolyte solution collapses due to a physical stimulation such as shock, resulting in the formation of a Nano bubble (100 nm in diameter) under high temperature and pressure [12]. oxidation ability of NBW3 is preserved as aqueous ozone for more than 6 months when sheltered from UV radiation. Because of its bactericidal efficiency and ease of use, NBW3 is utilised as a supplementary antiseptic in periodontal therapy.

### Advantages of topical ozone therapy:

There is always the possibility of antibiotic resistance developing. Pathogens, on the other hand, are unable to resist ozone's oxidative difficulties. Furthermore, ozone has been shown to directly inactivate bacterial toxins, whereas antibiotics do not [13]. Toxins have a significant role in bacterial tissue damage.

### Biocompatibility of ozone:

Gaseous and aqueous ozone were compared to recognised antiseptics chlorhexidine digluconate (CHX) 0.2 percent, sodium hypochlorite (NaOCl) 5.25 percent, 2.25 percent, and hydrogen peroxide H 2 O 2 3 percent in a research on human oral epithelial (BHY) and gingival fibroblast (HGF-1) cells [13]. The biocompatibility of the investigated antiseptics was greatest with aqueous ozone. When L-929 mouse fibroblasts were treated with ozonated water, their metabolic activity was high, but it was dramatically reduced when the cells were treated with 2.5 percent NaOCl. Irrigation of the root surfaces of avulsed teeth had no influence on the growth of periodontal ligament cells. Another research found that odontoblastic cells reacted to bacterial lipopolysaccharides with inflammatory responses (LPS). LPS-induced inflammatory responses were improved by using ozonated water [13].

## Dental applications:

### Ozone in dental caries:

Caries in the mouth is induced by an ecological niche of cariogenic organisms. The usefulness of ozone in the treatment of carious lesions has received a lot of attention in the literature [14]. This is due not just to ozone's antimicrobial characteristics, but also to the fact that ozone converts the cariogenic bacteria's pyruvic acid to acetate and carbon dioxide [15].

### Ozone in management of pit and fissure caries:

Deep pits and fissures are difficult to clean, making them more prone to food lodgement and bacterial development. In such circumstances, ozone treatment has proven to be quite helpful. Prior to ozone therapy, it is advised that the fissures be cleaned. This allows ozone to easily reach the caries. Following the ozone treatment, it is recommended that a remineralizing agent be used and the clean fissures be sealed [15]. The smear layer is removed by ozone, leaving exposed dentin that has been occluded by the remineralizing chemical. Over a three-month period, Huth et al. found that ozone administration dramatically decreased non-cavitated early fissure caries in individuals at high caries risk [16].

### Ozone in management of root caries:

In situations of superficial lesions, ozone is the most effective since it has an increased capacity to penetrate lesions up to 1mm deep. The ozone device must be be utilised properly, with the ozone cap held directly on the caries lesion to allow the ozone to penetrate the decay and biofilm. Simply applying ozone therapy to treat a cavitated 4mm deep root caries lesion next to the gingival edge is unlikely to be sufficient. To deal with a condition like this, the exterior caries must be removed first, leaving roughly 1 mm of caries on the cavity floor [16]. The use of ozone, followed by regular restoration, is then recommended. Rather than being viewed as a replacement for existing treatment and prevention strategies, ozone should be viewed as a supplement.

### Ozone therapy in hypersensitive teeth:

Tooth structure loss caused by a variety of processes such as attrition, abrasion, erosion, and stress from occlusion can wear away enamel and dentin, resulting in hypersensitivity [17]. Ozone treatment has been shown to successfully reduce sensitivity of exposed enamel and dentin, as well as root sensitivity [18]. The use of ozone for 40–60 seconds has been reported to alleviate pain in these sensitive teeth instantaneously. Ozone causes the smear layer to be removed, as well as the opening and widening of the dentinal tubules [19]. Calcium and fluoride ions enter the dentinal tubules quickly, freely, and fully when a remineralizing agent is applied, inhibiting fluid exchange from these tubules. As a result, ozone treatment causes sensitivity to be terminated in seconds and lasts longer than traditional approaches [20].

## Ozone in mucositis:

Chemotherapy and radiation are commonly used in individuals with carcinomatous lesions, and they almost always result in mucositis [21]. In instances of mucositis, ozone treatment in both aqueous and gaseous forms has shown significant outcomes, allowing the patient to eat normally and improving the patient's quality of life throughout oncological therapeutic operations.

## Ozone in wound healing and osseointegration:

Ozone therapy has been utilised in the treatment of severe jaw infections and osteotomies. Ozone promotes wound healing, improves erythrocyte characteristics, and enables oxygen delivery to tissues. This results in vasodilation, which increases blood flow to the ischemic areas. As a result, it can be utilised successfully in situations of poor wound healing following surgical procedures such as tooth extractions or implant dentistry. [20] Kazancioglu et al. studied the effects of ozone treatment on postoperative pain, oedema, and trismus after third molar surgery and found that it successfully decreased postoperative discomfort.

## Ozone therapy in periodontitis and peri-implantitis:

The impact of ozonated water irrigation on the proliferation of cells in the periodontal ligament adhering to the root surfaces of avulsed teeth was studied by Ebensberger et al. They found that irrigating avulsed teeth with ozonized water for 2 minutes resulted in efficient mechanical washing and root surface decontamination, with no negative effects on periodontal cells on the tooth surface [21].

Dental plaque samples were treated with 4 mL of ozonated water for 10 seconds in a research by Nagayoshi et al. They discovered that ozonated water killed gram-positive and gram-negative oral bacteria, as well as oral Candida albicans [21]. This is due to the fact that it has the ability to suppress pathogenic microorganisms in tooth plaque. In patients with advanced periodontitis, Ramzy et al. employed 150 mL of ozonized water to irrigate periodontal pockets for 5-10 minutes once weekly for four weeks. In terms of pocket depth, plaque index, gingival index, and bacterial count, he made remarkable progress. Ozone has also been utilised as a pre-treatment rinse to irrigate periodontal pockets before scaling and root planing procedures [21]. In individuals with acute necrotizing ulcerative gingivitis, ozonated oils have been shown to be beneficial. Karapetian et al. looked at the cases of patients with peri-implantitis. They examined the efficacy of traditional, surgical, and ozone therapy treatments for periimplantitis. The key obstacle, they say, appears to be decontaminating the implant surface and surrounding tissue, as well as preventing recolonization with periodontal pathogenic bacteria. The ozone-treated patient group showed the most efficient bacterial decrease [22].

## Implant surface decontamination procedure:

[1] After non-surgical treatment, mechanical debridement with titanium curettes is performed at the peri implantitissites [22].

[2] The ozone delivery system, which includes a variety of ozone generators, will be utilised to produce ozone at a preset concentration of 2,100 ppm via a hand piece.

[3] Using a sterile specifically made perio tip, ozone was administered at 6 sites circumferentially for 30 seconds at each point mesial, midbuccal, midlingual, and distal [22].

[4] The perio tip was stopped 1mm short of the pocket depth, and ozone [80 percent oxygen] was administered for 30 seconds three times a week for one week.

[5] The decontamination procedures were done three times on the treatment's start date, then two days and four days afterwards.

## Ozone toxicity:

Ozone is not hazardous when delivered at 0.05 ppm for 8 hours. During ozone treatment, the maximum concentration of ozone in the oral cavity is 0.01 ppm. Cough, nausea, vomiting, headache, epiphora, rhinitis, upper respiratory irritation, shortness of breath, and heart-related disorders are some of the adverse effects [23].

## Contraindications:

Acute alcohol intoxication, pregnancy, severe anaemia, recent myocardial infarction, hyperthyroidism, active haemorrhage and thrombocytopenia are known [23].

## Ozone intoxication:

[1] Patient must be placed in supine position

[2] Vitamin E

[3] Ascorbic acid

[4] Inhale humid oxygen

[5] N-Acetylcysteine.

## Conclusion:

Compared to standard treatment modalities like antibiotics and disinfectants, ozone therapy is very cost-effective; it will significantly reduce both medical costs and invalidity. The practise of dentistry is changing as a result of the introduction of contemporary science. Ozone therapy has proven to be more helpful than current conventional therapeutic techniques for dental treatment that are minimally invasive and conservative. [23] The explanation of ozone's chemical processes is also beneficial to dental practise. When patients are treated with ozone therapy, the treatment duration is drastically reduced, and the bacterial count is more precisely eradicated [23]. The procedure is painless and has little side effects, increasing the patient's tolerability and fulfilment with minimal adverse effects. Contraindications of this controversial method should not be forgotten. Further research is needed to regulate indications and treatment procedures of ozone therapy.
